# Telemedicine for stroke: a Brazilian success story

**DOI:** 10.1055/s-0045-1811724

**Published:** 2026-07-16

**Authors:** Guilherme Nobre Nogueira

**Affiliations:** 1 Universidade Federal do Ceará, Faculdade de Medicina, Departamento de Medicina Clínica, Fortaleza CE, Brazil.

Dear Editor,

I found the article by Ricarte et al. 1 to be incredibly engaging, as it provides a thorough and timely look at how a telemedicine-based stroke care network has been put into action. The authors present some compelling evidence showing that this initiative has greatly improved access to reperfusion therapy and helped bridge the geographical gaps in stroke management, especially in areas with limited resources.

The study deserves praise for its solid methodology and clear impact, with thrombolysis rates exceeding national averages and an impressive average door-to-computed tomography (CT) time of just 23 minutes. It really highlights the significance of regionalization and following established protocols in care. That said, a more in-depth cost-effectiveness analysis would have strengthened the study, especially since scalability is a key point. It would also have been beneficial for the article to dive deeper into the challenges surrounding mechanical thrombectomy, which remains surprisingly low (0.5%), even with the program's success in intravenous thrombolysis. Determining whether this low rate is due to logistical, infrastructural, or policy issues would add valuable insights and help guide future expansions.

Recent studies further underscore the importance of this topic, as shown in Table 1 . For example, Silva et al. conducted a nationwide study in Brazil and showed that ongoing telestroke networks not only boost thrombolysis rates but also enhance long-term functional outcomes when paired with continuous education and centralized feedback mechanisms. 2 Similarly, Grunwald et al. highlighted how artificial intelligence (AI)-based triage tools in telestroke systems can speed up decision-making and cut down door-to-needle times by as much as 20% in real-world situations. 3 Another noteworthy study, by Puhr et al., found that remote thrombolysis through mobile stroke units equipped with CT capabilities significantly improved stroke care in rural areas, although it did come with higher operational costs. 4 Finally, Duarte et al. analyzed equity in telestroke systems and highlighted that cultural and linguistic adaptation of interfaces and communication platforms further enhances patient engagement and outcomes in diverse regions. 5

**Table 1:** Articles on stroke unit availability

Study/Location	Thrombolysis rate (%)	Mean door-to-needle time (minutes)	Stroke unit availability	Highlighted additional strategy
Ricarte et al. 1 /Brazil	31.8 (ischemic)/15.6 (overall)	67.1	Partial (1 unit)	Telemedicine across 6 regional stroke centers
Silva et al. 2 /Brazil	28.4	63	Yes	Continuous education + centralized feedback
Grunwald et al. 3 /Multicenter	33.0	53	Yes	AI-based triage system
Puhr et al. 4 /Europe	36.2	48	Yes (mobile)	Mobile stroke unit with onboard CT
Duarte et al. 5 /Brazil	29.5	59	Yes	Culturally-adapted telestroke interface

Abbreviations: AI, artificial intelligence; CT, computed tomography.

When looked at together, these studies support the main idea of the article, which is that telemedicine is an important tool to increase accessibility to acute stroke therapy. However, they also indicate that the next steps of the Piauí initiative should include AI triage, training programs, mobile CT strategies, and cultural tailoring to make it more efficient and welcoming.

In conclusion, the study by Ricarte et al. 1 gives us hope that telemedicine can help fill in the gaps in stroke care that have existed for a long time in areas that don't have the necessary medical support. The project in Piauí is a big step forward but using lessons from recent global advances, like AI-driven triage, mobile CT units, and culturally adaptive systems, can lead to better outcomes and fairness. Continued investment, monitoring, and innovation will be the key to ensuring that telemedicine fulfills its potential as a transformative tool in global stroke care.
